# Identification and validation of potential diagnostic signature and immune cell infiltration for NAFLD based on cuproptosis-related genes by bioinformatics analysis and machine learning

**DOI:** 10.3389/fimmu.2023.1251750

**Published:** 2023-09-26

**Authors:** Guoqing Ouyang, Zhan Wu, Zhipeng Liu, Guandong Pan, Yong Wang, Jing Liu, Jixu Guo, Tao Liu, Guozhen Huang, Yonglian Zeng, Zaiwa Wei, Songqing He, Guandou Yuan

**Affiliations:** ^1^ Division of Hepatobiliary Surgery, The First Affiliated Hospital of Guangxi Medical University, Nanning, Guangxi, China; ^2^ Key Laboratory of Early Prevention and Treatment for Regional High Frequency Tumor (Guangxi Medical University), Ministry of Education, Nanning, Guangxi, China; ^3^ Guangxi Key Laboratory of Immunology and Metabolism for Liver Diseases, Guangxi Medical University, Nanning, Guangxi, China; ^4^ Liuzhou Key Laboratory of Liver Cancer Research, Liuzhou People’s Hospital, Liuzhou, Guangxi, China; ^5^ Liuzhou Hepatobiliary and Pancreatic Diseases Precision Diagnosis Research Center of Engineering Technology, Liuzhou People’s Hospital by Liuzhou Science and Technology Bureau, Liuzhou, Guangxi, China; ^6^ Department of General Surgery, Luzhai People’s Hospital, Liuzhou, Guangxi, China

**Keywords:** nonalcoholic fatty liver disease, cuproptosis, immune infiltration, machine learning, immune microenvironment

## Abstract

**Background and aims:**

Cuproptosis has been identified as a key player in the development of several diseases. In this study, we investigate the potential role of cuproptosis-related genes in the pathogenesis of nonalcoholic fatty liver disease (NAFLD).

**Method:**

The gene expression profiles of NAFLD were obtained from the Gene Expression Omnibus database. Differential expression of cuproptosis-related genes (CRGs) were determined between NAFLD and normal tissues. Protein–protein interaction, correlation, and function enrichment analyses were performed. Machine learning was used to identify hub genes. Immune infiltration was analyzed in both NAFLD patients and controls. Quantitative real-time PCR was employed to validate the expression of hub genes.

**Results:**

Four datasets containing 115 NAFLD and 106 control samples were included for bioinformatics analysis. Three hub CRGs (NFE2L2, DLD, and POLD1) were identified through the intersection of three machine learning algorithms. The receiver operating characteristic curve was plotted based on these three marker genes, and the area under the curve (AUC) value was 0.704. In the external GSE135251 dataset, the AUC value of the three key genes was as high as 0.970. Further nomogram, decision curve, calibration curve analyses also confirmed the diagnostic predictive efficacy. Gene set enrichment analysis and gene set variation analysis showed these three marker genes involved in multiple pathways that are related to the progression of NAFLD. CIBERSORT and single-sample gene set enrichment analysis indicated that their expression levels in macrophages, mast cells, NK cells, Treg cells, resting dendritic cells, and tumor-infiltrating lymphocytes were higher in NAFLD compared with control liver samples. The ceRNA network demonstrated a complex regulatory relationship between the three hub genes. The mRNA level of these hub genes were further confirmed in a mouse NAFLD liver samples.

**Conclusion:**

Our study comprehensively demonstrated the relationship between NAFLD and cuproptosis, developed a promising diagnostic model, and provided potential targets for NAFLD treatment and new insights for exploring the mechanism for NAFLD.

## Introduction

Nonalcoholic fatty liver disease (NAFLD), a common liver disorder worldwide, is characterized by the accumulation of hepatic fat without excessive alcohol consumption or other damage factors that cause chronic liver dysfunction (1). NAFLD includes a spectrum of conditions ranging from simple steatosis and nonalcoholic steatohepatitis (NASH) to cirrhosis and even hepatocellular carcinoma (2). In recent years, NAFLD has emerged as a serious global health concern, affecting nearly 30% of the general population and leading to 172,329.57 incidences worldwide in 2019 (3, 4). The pathogenesis of NAFLD is usually explained with the most well-recognized theory of the “two-hit” hypothesis (5). The “first hit” is intrahepatic fat deposition, and the “second hit” includes oxidative stress, inflammation, and mitochondrial dysfunction, resulting in liver injury and fibrosis (6, 7). Although a recent “multiple-hit” hypothesis has been proposed, it still does not fully explain the intrinsic mechanisms of NAFLD (5).

Copper is an important cofactor in the biological process of cells in the organism. Copper imbalance may lead to various diseases, such as Wilson’s disease, blood diseases, and cancer (8, 9). Excess copper can lead to cell death, and copper deficiency may weaken the functions of the copper-binding enzymes (10). However, the mechanism of copper leading to cell death is still a boundary to explore. Recently, Tsvetkov et al. revealed a novel mechanism of the copper-induced form of cell death regulation, coined as cuproptosis, which is independent of other cell death processes, including autophagy, proptosis, apoptosis, and ferroptosis (11). Cuproptosis is defined as the copper imbalance accumulation and binding to tricarboxylic acid (TCA) cycle proteins, resulting in lipoylated component abnormal aggregation and iron–sulfur cluster protein loss, leading to proteotoxic stress and ultimately causing cell death (11).

Copper is primarily stored in the liver, and excess endogenous copper is mainly eliminated through biliary excretion (9, 12). Wilson’s disease is an example of copper overload in the liver caused by a mutation in the ATP7B gene (13), which was reported to be potentially mediated by cuproptosis (9). Previous studies indicated that low copper concentration may promote the development of NAFLD (14). Excess serum copper activates autophagy, oxidative, and Nrf2 signaling, and up-regulates lipid metabolism and lipogenesis, which can induce the occurrence of NAFLD (15). In addition, some studies have found excess copper accumulation in the end-stage NAFLD patients (16). Furthermore, accumulating evidence suggests that copper plays a role in regulating the immune system (17, 18). However, as a novel discovered form of program regulated cell death, the potential regulatory mechanisms of cuproptosis in NAFLD are not yet understood, and the potential role of cuproptosis as a treatment target for NAFLD requires further study. In this study, we analyzed differentially expressed cuproptosis-related genes (CRGs) and their immune characteristics between 115 NAFLD and 106 control cases. Machine learning algorithms were used to find the hub genes to help predict the diagnosis. The predictive model was validated using a nomogram, decision curve analysis (DCA), calibration curve, and receiver operating characteristic (ROC) curve. In addition, the relationship between hub CRGs and immune infiltration was explored. Finally, potential target drugs and ceRNA networks were also established in our study.

## Materials and methods

### Data collection and processing

The transcriptome profiling data for NAFLD and control (non-NAFLD) samples were downloaded from five datasets in the Gene Expression Omnibus (GEO) database, including GSE48452, GSE135251, GSE66676, GSE89632, and GSE63067. Specifically, GSE48452 (based on the GPL11532 platform) contained 14 healthy control, 27 healthy obese, 14 steatosis, and 18 NASH samples. GSE63067 (GPL570 platform) included 2 steatosis, 9 NASH, and 7 healthy control cases. GSE66676 (GSE6244 platform) contained 26 steatosis, 7 NASH, and 34 healthy control samples. GSE89632 (GSE14951 platform) contained 20 steatosis, 19 NASH, and 24 healthy control samples. The GSE135251 dataset (53 steatosis, 153 NASH and 10 control samples) was used to validate the expression of hub genes. The combat function of the “sva” package was used to eliminate the batch effects and latent unknown variables (19).

**Figure 1:**
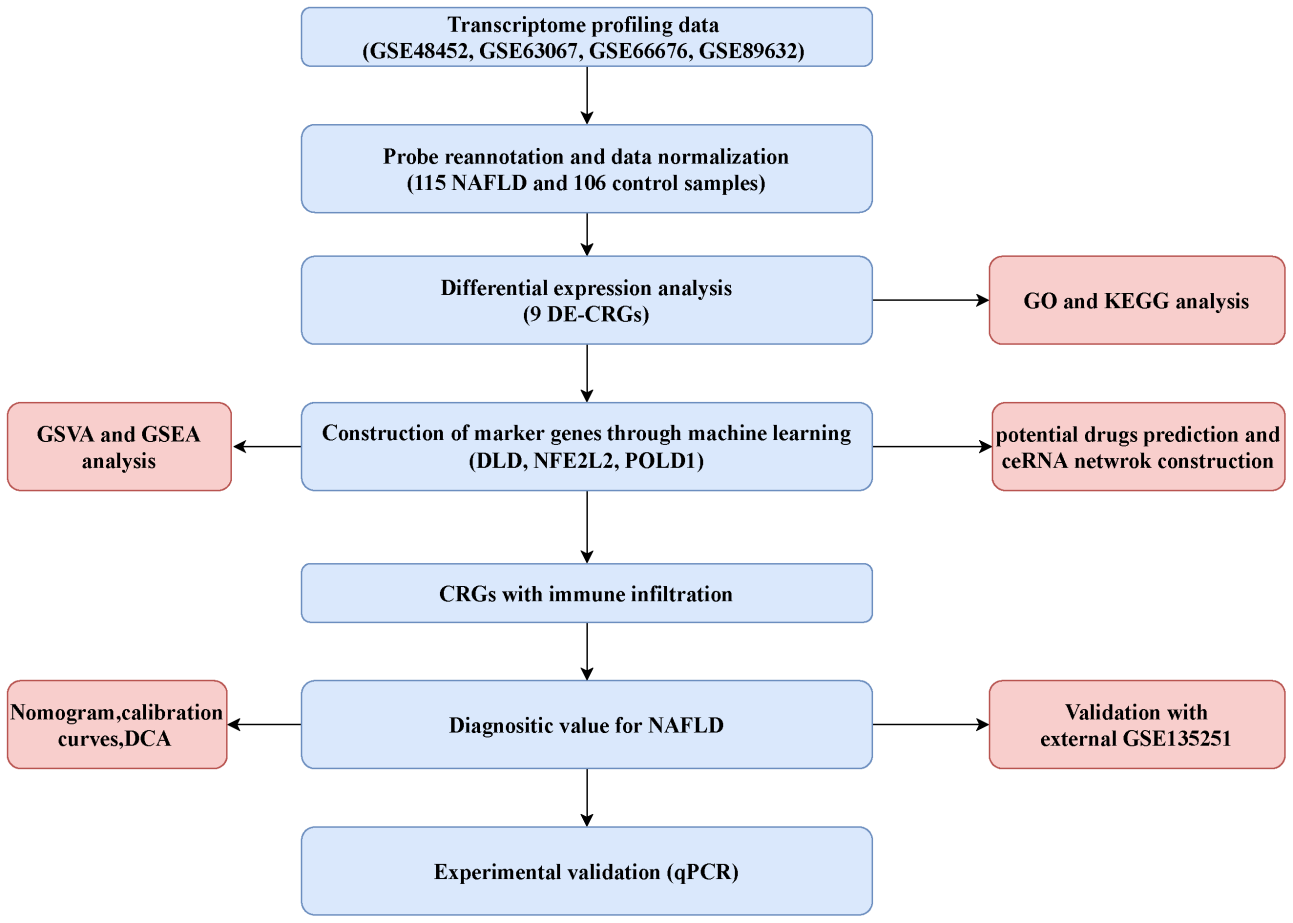
Flowchart of the present study.

After merging the data of GSE48452, GSE66676, GSE89632, and GSE63067, the merged datasets included 115 NAFLD cases and 106 control cases. A total of 38 CRGs were retrieved from previous literature (ATP7B, CDKN2A, DLD, DPYD, FDX1, GLRX5, GLS, ISCA2, LIPT1, MTF1, NDUFA1, NDUFA8, NDUFB10, NDUFB2, NDUFB6, NDUFC1, NDUFC2, NDUFV2, PDHA1, PLAT, POLD1, PPAT, SLC31A1, SDHB, TIMMDC1, DLAT, GCSH, DBT, DLST, LIAS, LIPM, LIPA, LIPT2, PDHB, ACO2, NLRP3, ATP7A, and NFE2L2) (20, 21). The flowchart of the present study is shown in **Figure 1**.

### Differential gene expression analysis of CRGs

The Wilcoxon signed-rank test was used to detect differentially expressed genes (DEGs) of CRGs between the NAFLD and control groups. A boxplot of DEGs of 38 CRGs was constructed using the “ggpubr” R package. The results were visualized as volcano and heatmap plot using the “ggplot2” and “pheatmap” R packages. The intersection of DEGs related to CRGs was created using the “VennDiagram” R package and defined as DEG-CRGs for subsequent analysis. Significant differences were considered when p < 0.05.

### Correlation analysis and PPI network construction

The landscape of the 23 chromosomes and a heatmap of 9 DE-CRGs were generated using the R package “RCircos” and “heatmap,” respectively. A correlation Circos plot based on Pearson’s correlation analysis between DEG-CRGs was created using the “circlize” package. Protein–protein interaction (PPI) network of nine DE-CRGs was constructed using the STRING database (https://string-db.org/). A medium confidence of 0.4 was set for PPI analysis.

### Function enrichment analysis of DEG-CRGs

Gene Ontology (GO), including biological process, molecular function (MF), and cellular component, and Kyoto Encyclopedia of Genes and Genomes (KEGG) pathway analyses on nine DEG-CRGs were performed using “clusterProfiler” R package and visualized using the R “enrichplot” package. A significant enrichment threshold was set at p < 0.05.

### Construction of CRG diagnostic model

The merged data of GSE48452, GSE66676, GSE89632, and GSE63067 were used as training set, while GSE135251 dataset was used as the validation dataset for machine learning model. The random forest (RF) algorithms, support vector machine–recursive feature elimination (SVM–RFE), and least absolute shrinkage and selection operator (LASSO) regression were used to identify the most powerful hub gene for NAFLD prognosis. LASSO regression was implemented with the “glmnet” package in R to the selected linear model, reducing data dimension and keeping the valuable variables (22, 23). The minimum lambda value was set as the optimal value to build the model. SVM–RFE is a supervised machine learning model to distinguish between positive and negative instances by deleting the feature vector created by SVM. The R “e1071” package was used to create the SVM-RFE model to filter the best variable genes (24, 25). The SVM-RFE method was utilized to determine the optimal variables by searching for the point corresponding to the minimum cross-validation error. The RF model is an ensemble machine learning method used to determine the optimal number of variables using various independent decision trees (26). RF was performed using the “randomForest” R package and “ntree” set at 500. The intersection was used to select the most powerful hub genes in the present study derived from RF, LASSO algorithms, and SVM–RFE.

### ROC and nomogram model construction

The diagnostic value of the marker genes was evaluated with time-dependent ROCs, assessing the area under the curve (AUC), specificity, and sensitivity. The R “pROC” package was used to perform the ROC curve analysis (27). To further validate the hub genes, the external GSE135251 dataset was used to verify the diagnostic ability of the diagnostic model.

A nomogram model was constructed to predict the occurrence of NAFLD using the R “rms” package. Each hub gene owns a unique score, and the “total points” is the sum scores of the aforementioned predictors. The calibration curve was employed to assess the predictive power. In addition, clinical impact curves and decision curves were created to estimate the clinical utility of this model.

### Gene set enrichment analysis and gene set variation analysis

To identify the potential function of hub genes, we used the Gene Set Enrichment Analysis (GSEA) function of the R “clusterProfiler” package[ (28)]. We selected the reference KEGG gene set (c2.cp.kegg.symbols.gmt) from the Molecular Signatures Database. Statistical significance was defined as p < 0.05 for enrichment analysis.

To illustrate the differentially enriched gene sets between high- and low-expression subtypes based on the expression levels of the hub genes, gene set variation analysis (GSVA) was performed using the “GSVA” R package. The R “limma” package was used to discover the differential expression pathways by comparing GSVA scores between low- and high-expression subtypes.

### Evaluating the immune infiltration

The CIBERSORT algorithm was used to estimate the fractions of 22 types of human immune cells in each sample from the merged dataset (29). An accurate immune cell fraction was defined as having a p value <0.05. For each sample, the sum fractions of the 22 immune cells equal 1.

To assess the enrichment score of infiltrating immune cells and immune-related functions in each sample, single-sample gene set enrichment analysis (ssGSEA) was performed using the R “GSVA” package. The reference gene set was downloaded from the ImmPort database (http://www.immport.org). The correlation between the hub gene and immune score was determined using Spearman’s correlation analysis. The difference in enrichment scores of immune cells and immune-related function was estimated using the Wilcoxon test. The composition of the enrichment score between NAFLD and the control group was visualized by a boxplot.

### Identification of potential small molecule drugs

Drug–gene interaction databases (DGIdb, https://dgidb.genome.wustl.edu/) are online databases that provide drug–gene/protein interaction information collected from many sources, such as The Druggable Genome and Therapeutic Targets Database (30). Additionally, the Drug Signatures Database (DSigDB) was used to predict candidate drugs associated with the three hub genes, with access to DSigDB through the Enrichr website (https://amp.pharm.mssm.edu/Enrichr/). To predict potential drugs that may interact with the marker genes, both DGIdb and DSigDB were used. The drug–gene network of DGIdb was visualized using Cytoscape software.

### ceRNA network construction

Based on the three hub genes, the miRDB (http://www.mirdb.org/) and TargetScan (https://www.targetscan.org/vert_80/) databases were used to predict miRNA–mRNA interactions. SpongeScan (http://spongescan.rc.ufl.edu/) was used to integrate evidence for direct interaction between the predicted miRNA and lncRNA. Finally, a ceRNA network of mRNA–miRNA–lncRNA was established and visualized through Cytoscape software (version 3.9.0).

### NAFLD mouse model construction and histological procedure

Eight-week-old male C57BL/6J mice were fed a 60% high-fat diet and a 10% fat diet (control group) for 12 weeks. Eight wild-type mice and eight NAFLD mice were enrolled in this study. The serum and pathological tests were used to confirm the successful NAFLD model. Fresh liver samples were fixed with 4% formaldehyde for paraffin embedding, and 4-μm sections were used for the H&E staining according to the manufacturer’s instructions. As for oil red O staining, frozen 8-μm sections were fixed with 10% calcium formaldehyde, then washed with 60% isopropanol, and finally stained with oil red O solution for 30 min at 37°C. All animal experiments were approved by the Animal Care and Use Committee of Guangxi Medical University.

### RNA extraction, quantitative real-time PCR

Total RNA was extracted from NAFLD and wild-type mouse tissues using TRIzol reagent (Thermo Fisher Scientific, USA). The RNA was reverse transcribed using the PrimeScript™ RT reagent Kit (Takara, Japan), and quantitative real-time PCR (qRT-PCR) was performed using the FX Connect system (Bio-Rad, USA) and SYBR^®^ Green Supermix (Bio-Rad, USA). Hub genes expression levels were analyzed using 2^−ΔΔCT^, and the outcomes were demonstrated using GAPDH as an internal control. The primers used in the qRT-PCR assays are listed in **Supplementary Table S1**.

### Statistical analyses

The statistical and data analyses were performed utilizing R software (version 4.2.1). Continuous data are expressed as mean ± standard deviation, ensuring adherence to previous studies while avoiding redundancy. To compare two groups, the Student’s t-test was applied for normally distributed variables, while the Wilcoxon rank-sum test was utilized for non-normally distributed variables, preventing duplication with existing publications. A two-tailed p-value of less than 0.05 was considered statistically significant, maintaining consistency with accepted significance levels. Significance levels were denoted as ***, **, and * for p-values less than 0.001, 0.01, and 0.05, respectively. The R codes is available at https://github.com/ouyan1990/NAFLD-cuproptosis.

## Results

### Identification of cuproptosis-related genes involved in NAFLD

**Figure 2:**
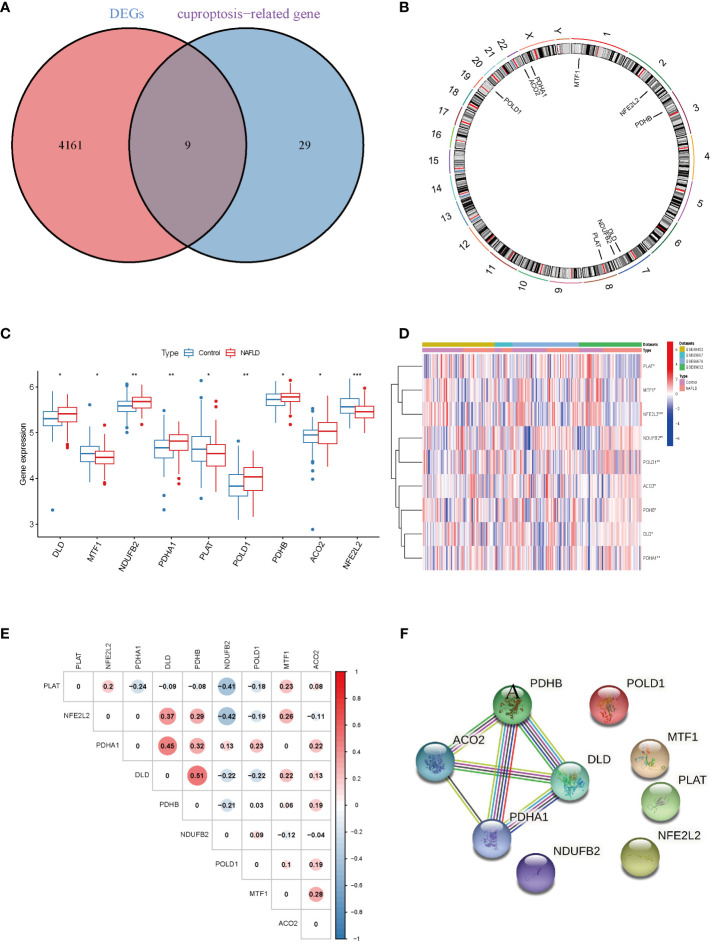
Identification of cuproptosis-related differentially. Expressed genes in NAFLD. **(A)** Venn diagram showed the intersection of genes between DEGs and cuproptosis-related genes. **(B)** The locations of the 9 DE-CRGs on 23 chromosomes. **(C)** Boxplots showed the differential expression of CRGs between NAFLD and control samples. **(D)** The expression patterns of 9 DE-CRGs were shown in the heatmap. **(E)** Correlation analysis of 9 DE-CRGs. Red and Blue colors represent positive and negative correlations, respectively. **(F)** Gene relationship network diagram of 9 DE-CRGs. p values were showed as: *, p < 0.05; **, p < 0.01; ***, p < 0.001. DEG, differential expression genes; DE-CRGs, differentially expressed cuproptosis-related genes.

Four datasets (GSE48452, GSE63067, GSE66676, and GSE89632), including 115 NAFLD samples and 106 control samples, were merged and batch-normalized. A total of 4,170 DEGs were identified using the “limma” package with *p* < 0.05, of which 2,141 were downregulated and 2,129 were upregulated. The volcano plots of GSE48452, GSE66676, GSE89632, GSE63067 and merged data were shown in **Figure S1**. The gene expression patterns of 4,170 DEGs are presented in the heatmap (**Figure S2**) The GO and KEGG analyses of the total DEGs were presented in **Figure S3**. Additionally, by overlapping the 4,170 DEGs with 38 CRGs, 9 DE-CRGs (MTF1, POLD1, NFE2L2, ACO2, PDHB, DLD, NDUFB2, PLAT, and PDHA1) that differed significantly between the NAFLD and control groups were identified (**Figure 2A**). The chromosomal locations of the nine DE-CRGs were depicted on a loop graph (**Figure 2B**). We found that DLD, NDUFB2, PDHA1, POLD1, PDHB, and ACO2 were upregulated in NAFLD, while MTF1, PLAT, and NFE2L2 were downregulated (**Figures 2C, D**). The correlations between the nine DE-CRGs were depicted in **Figure 2E**. DLD was positively associated with PDHB, MTF1, and ACO2 was negatively correlated with NDUFB2 and POLD1. To investigate the potential crosstalk between these nine DE-CRGs, PPI analyses were performed using STRING, which were presented in **Figure 2F**.

### Enrichment analysis of the differential CRGs

**Figure 3:**
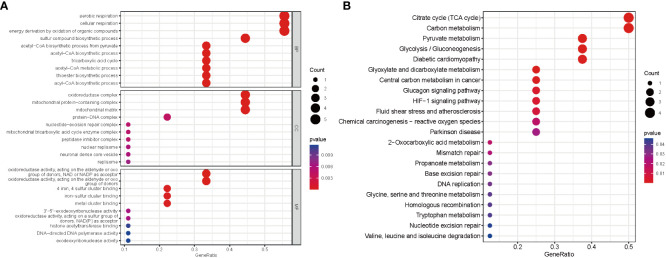
Functional analyses for the DE‐CRGs. **(A)** Bubble diagrams of the GO enrichment analysis of 9 DE-CRGs. **(B)** Bubble diagrams of the KEGG enrichment analysis of 9 DE-CRGs. DE-CRGs, differentially expressed cuproptosis-related genes. GO, Gene Ontology; BP, Biological process; CC, Cellular component; MF, Molecular function; KEGG, Kyoto Encyclopedia of Genes and Genomes.

On the basis of these nine DE-CRGs, we performed GO and KEGG enrichment analyses to illustrate the biological function and pathways using “ClusterProfler” packages. The biological process analysis indicated enriched in cellular respiration, aerobic respiration, energy derivation by oxidation of organic compounds, and the TCA cycle. The cellular component analysis was significantly involved in the oxidoreductase complex, mitochondrial protein–containing complex, mitochondrial matrix, and protein–DNA complex. MF was largely related to oxidoreductase activity, acting on the aldehyde or oxo group of donors, iron–sulfur cluster binding, and metal cluster binding (**Figure 3A**). Interestingly, the KEGG pathway analysis revealed that the nine DE-CRGs were notably associated with the citrate cycle (TCA cycle), carbon metabolism, pyruvate metabolism, and some other metabolic pathways similar to those revealed by GO analysis, such as acetyl-CoA metabolic process and sulfur compound biosynthetic process (**Figure 3B**).

### Construction of the diagnostic marker genes for NAFLD

**Figure 4:**
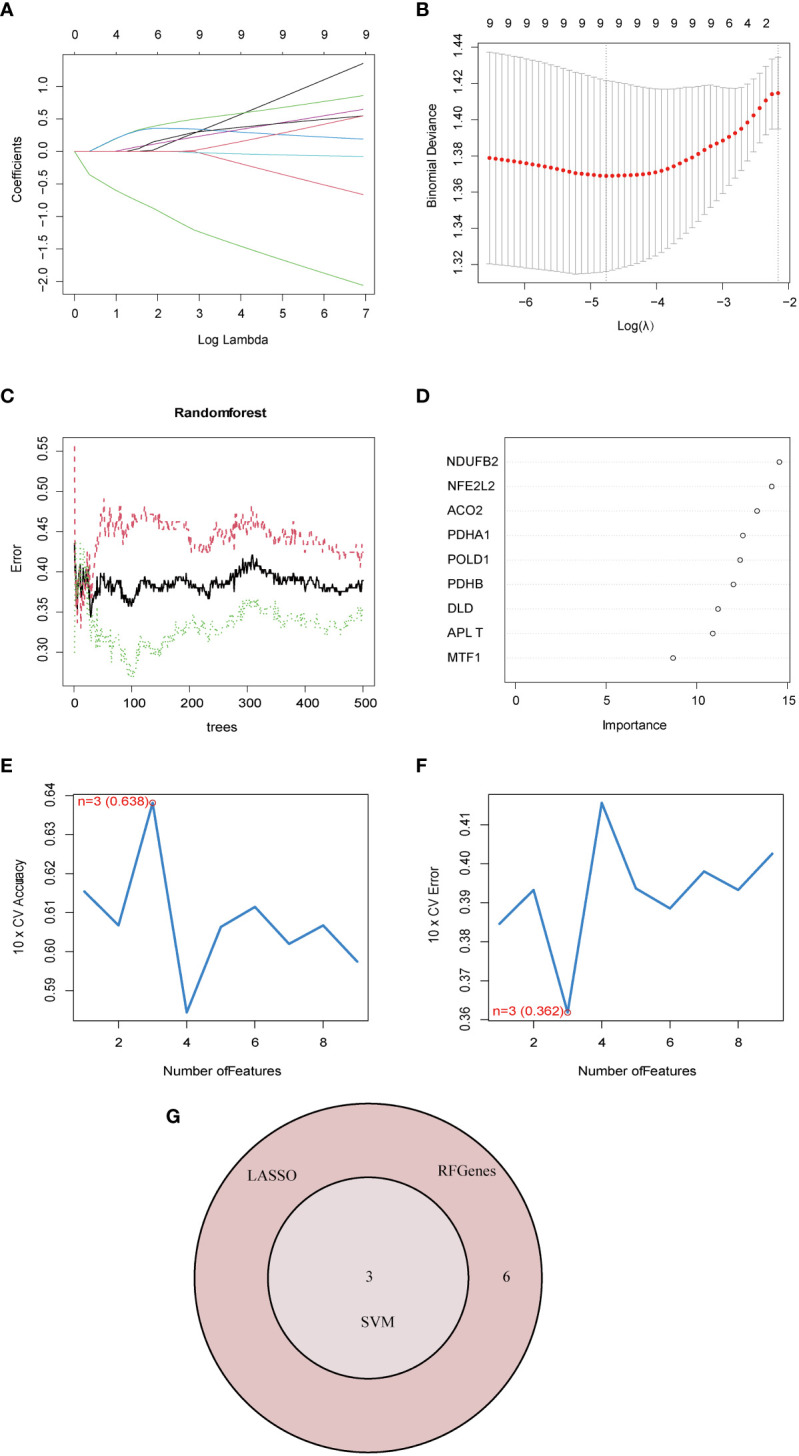
Identification of diagnostic marker genes for NAFLD through machine learning. **(A)** Ten-time cross-verification of adjusted parameter selection in the LASSO model. Each curve corresponds to one gene. **(B)** LASSO coefficient analysis. The solid vertical lines represent the partial likelihood deviance SE. The dotted vertical line is drawn at the optimal lambda. **(C)** Relationship between the number of random forest trees and error rates. The red line represents the error of the NAFLD group, the green line represents the error of the Control group, and the black line represents the total sample error. **(D)** The rank of genes in accordance with their relative importance. **(E)** The accuracy and **(F)** the error of the feature selection for the SVM-RFE algorithm. The peak of the curve is achieved at 3 genes with an accuracy of 63%, with the lowest cross-validation error is found in 3 gens and the values is 36.2%.**(G)** The Venn diagram shows the overlap of marker genes between LASSO, random forest, and SVM-RFE algorithms.

To account for the individual complexity and heterogeneity between NAFLD patients and control subjects, a LASSO regression and two proven machine learning models (SVM–RFE and RF) were used to identify candidate CRG regulators from the nine DE-CRGs, which could aid in the prediction of NAFLD diagnosis. Through the LASSO logistic regression algorithm, nine DG-CRGs were identified (**Figures 4A, B**). Concerning RF, nine DG-CRGs were selected, including NDUFB2, NFE2L2, ACO2, PDHA1, POLD1, PDHB, DLD, PLAT, and MTF1 (**Figures 4C, D**). As for SVM–RFE, the number of features was 3, which experienced the lowest classifier error and highest classifier accuracy (minimal error = 0.362, maximal accuracy = 0.638), including NFE2L2, DLD, and POLD1 (**Figures 4E, F**). The Cumulative residual distributions and ROC results of RF and SVM were shown in **Figure S4** and showed a higher sensitivity of prediction. The hub genes of LASSO, SVM–RFE, and RF were then intersected using a Venn diagram. Ultimately, three hub genes (NFE2L2, DLD, and POLD1) were identified for further analysis (**Figure 4G**).

### Validation of marker gene expression

**Figure 5:**
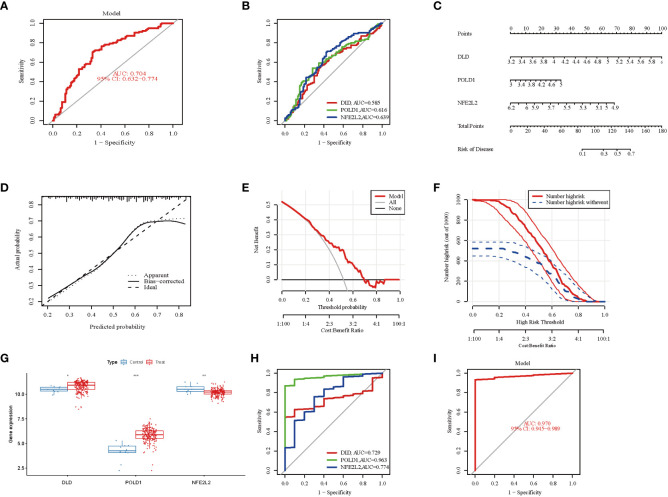
Validation of marker gene expression. **(A)** Logistic regression model to identify the AUC of NAFLD. The AUC value was 0.704(95%CI 0.632-0.774). **(B)** The ROC results for the 3 marker genes. The AUC value of DLD, POLD1, and NFE2L2 was 0.58, 0.616, 0.639, respectively. **(C)** Nomogram graph of the 3 marker genes. **(D)** Calibration curve displaying the diagnostic ability of the nomogram model. **(E)** DCA illustrating the predictive efficiency of Nomogram models. **(F)** The clinical impact curve showed a higher diagnostic ability of the nomogram model. **(G)** Boxplots indicating the three differentially expressed CRGs were significant alter between NAFLD and control samples in GSE135251. **(H)** The ROC results of 3 marker genes in GSE135251. The AUC value of DLD, POLD1, and NFE2L2 was 0.729, 0.963, 0.774, respectively. **(I)** ROC results of the 3-gene-based model based on 3-fold cross-validation in GSE135251. The AUC value as 0.970. AUC, area under curve; ROC, receiver operating characteristic; DCA, Decision curve analysis.

The ROC curve analysis demonstrated that the three-marker-gene signature had a high diagnostic value, with an AUC value of 0.704 (**Figure 5A**). ROC curves of three marker genes were generated to elucidate the predictive value of individual genes. **Figure 5B** illustrates the ROC results for these three genes, which are all greater than 0.5.

To further evaluate the predictive efficiency of these three hub genes, a nomogram model was constructed using the “rms” package for NAFLD patients based on DLD, POLD 1, and NFE2L2(**Figure 5C**). Each biomarker was assigned a score on the nomogram, followed by the prediction of NAFLD risk based on the cumulative score. The calibration curves suggested a relative link between predicted and actual probabilities (**Figure 5D**). DCA indicated that the nomogram model had significantly higher net benefits than the individual hub genes, suggesting a high level of accuracy and providing a foundation for physician decision-making (**Figure 5E**). The clinical impact curve also indicated a relatively high diagnostic ability of this nomogram model (**Figure 5F**).

Furthermore, the gene expression and ROC curve of the three hub genes were validated using the GSE135251 dataset. The results indicated that the expression of DLD and POLD1 was upregulated, whereas the expression of NFE2L2 was downregulated (**Figure 5G**). As presented in **Figure 5H**, the AUC values of ROC curves for all hub genes were greater than 0.72 in the GSE135251 dataset (DLD, AUC = 0.729; POLD1, AUC = 0.963; NFE2L2, AUC = 0.774). In addition, the AUC values of the three hub genes together were higher than those of the unique gene among them, suggesting a more powerful predictive ability (AUC = 0.970) (**Figure 5I**). These results suggest that the three marker genes may serve as diagnostic biomarkers for NAFLD.

### Profile of GSEA and GSVA

**Figure 6:**
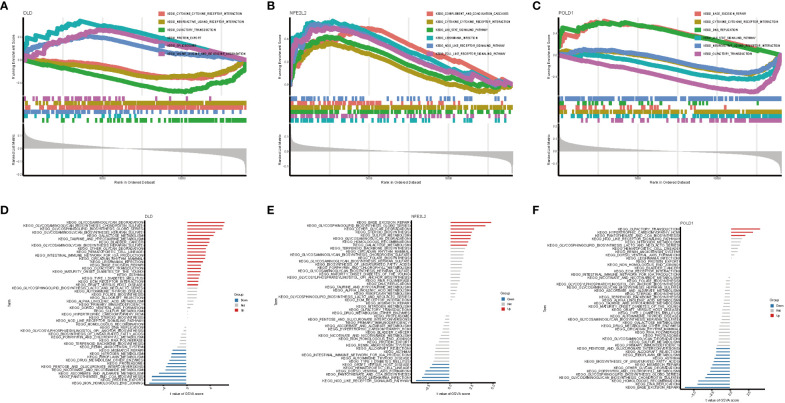
GSEA and GSVA analysis of three marker genes. The KEGG pathway enrichment analysis of **(A)** DLD, **(B)** NFE2L2 and **(C)** POLD1 were carried out by GSEA enrichment method, and the two items with the highest and lowest enrichment scores are visualized according to the arrangement of enrichment scores. The KEGG pathway enrichment analysis of **(D)** DLD, **(E)** NFE2L2 and **(F)** POLD1 was carried out by GSVA enrichment method, and the top 50 are visualized according to the enrichment score.

On the basis of the KEGG and GO pathways, we performed single-gene GSEA to identify the predominant signaling pathways for this model. GSEA of KEGG revealed that low expression of DLD and POLD1 was involved in cytokine–cytokine receptor interaction, whereas high expression of NFE2L2 participated in cytokine–cytokine receptor interaction (**Figures 6A–C**). Additionally, DLD and POLD1 were related to neuroactive ligand–receptor interaction and olfactory transduction (**Figures 6A, C**), whereas NFE2L2 and POLD1 were involved in the JAK–STAT signaling pathway. Furthermore, we found that DLD was associated with protein export, spliceosome, valine leucine and isoleucine degradation, and fatty acid metabolism (**Figure 6A**), whereas NFE2L2 was involved in complement and coagulation cascades, Leishmania infection, Toll-like receptor signaling pathway, and NOD-like receptor signaling pathway (**Figure 6B**). POLD1, in contrast, was related to base excision repair and DNA replication (**Figure 6C**). The GSEA result of GO enrichment is presented in **Figure S5**.

Next, GSVA was performed to detect the differentially active pathways between low- and high-expression subtypes on the basis of the expression level of three hub genes. Our analysis revealed that overexpression of DLD activated glycosaminoglycan degradation, galactose metabolism, hematopoietic cell lineage, intestinal immune network for IgA production, Leishmania infection, type I diabetes mellitus, base excision repair, and other pathways. In contrast, low expression of DLD was associated with NAFLD via active mismatch repair, protein export, nicotinate and nicotinamide metabolism, and proteasome pathway (**Figure 6D**). In the high expression of the NFE2L2 group, base excision repair, glycosaminoglycan degradation, and galactose metabolism pathways were active (**Figure 6E**). In contrast, only three pathways, namely olfactory transduction, hypertrophic cardiomyopathy, and pantothenate and CoA biosynthesis, were activated when POLD1 exhibited a high expression in NAFLD. However, low expression of POLD1 was enriched in more signaling pathways (**Figure 6F**). Proteasome pathway, base excision repair, pantothenate and CoA biosynthesis, which could be active by DLD, NFE2L2, and POLD1, plays a vital role in the development of NAFLD (31–33).

### Landscapes of immune infiltration between NAFLD and controls

**Figure 7:**
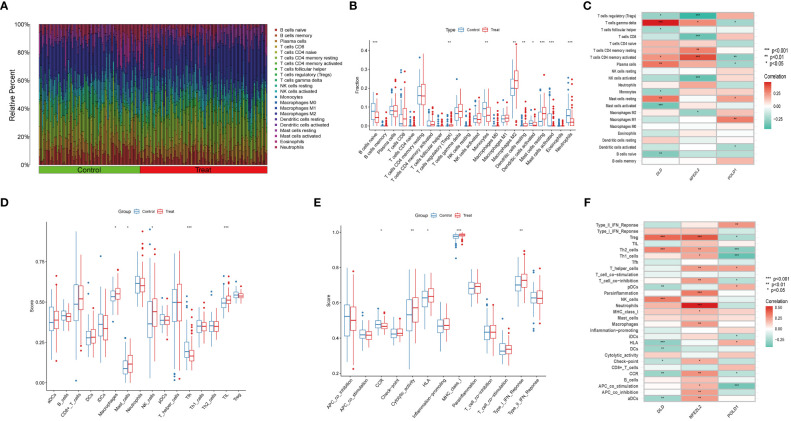
Immune Infiltration analysis between NAFLD and controls by CIBERSORT and ssGSEA algorithm. **(A)** The relative abundances of 22 infiltrated immune cells between NAFLD and control samples. **(B)** Boxplots indicated the differences in immune infiltrating between NAFLD and control samples. The results showed 9/22 of immune cells were significantly different between NAFLD and control group. **(C)** The correlation between 22 immune cells and three marker genes. Red and green colors represent positive and negative correlations, respectively. **(D, E)** Boxplots indicated the differences in immune cells and function between NAFLD and control samples. **(F)** The correlation between 29 immune cells and functions and three marker genes. Red and green colors represent positive and negative correlations, respectively. p values were showed as: *, p < 0.05; **, p < 0.01; ***, p < 0.001.

NAFLD is an inflammatory disease marked by the penetration o immune cells into plaques and hepatic lobule. Notably, cuproptosis has also been reported to play a regulatory role in the modulation of inflammation (9). In order to verify whether cuproptosis could promote NAFLD progression by mediating immune infiltration, we conducted CIBERSORT and ssGSEA analysis. We first used the CIBERSORT algorithm to assess the difference in the immune microenvironment between NAFLD and control samples. **Figure 7A** illustrates the proportion of 22 different immune cells’ expression between NAFLD and control samples. **Figure 7B** illustrates the expression of nine immune cell types exhibiting a significant difference between the two groups. Specifically, we found that macrophages M2, Treg cells, resting mast cells, and resting dendritic cells were more abundant in NAFLD patients, whereas monocytes, activated dendritic cells, activated mast cells, naive B cells, and neutrophils were less abundant (**Figure 7B**). Correlation analysis revealed a positive correlation between DLD and gamma-delta T cells and a negative correlation with activated mast cells (**Figure 7C**, **Table S2**). These results suggest that modifications in the immune microenvironment may contribute to the development of NAFLD.

Next, we used the ssGSEA algorithm to analyze the enrichment scores of distinct immune cells and functions or pathways between NAFLD and the control group. We found that macrophages, mast cells, NK cells, and tumor-infiltrating lymphocytes were significantly upregulated in NAFLD patients (**Figure 7D**). As for immune functions, the score of cytolytic activity, HLA, MHC class I, and type I IFN response were higher in NAFLD than in the control group (**Figure 7E**). **Figure 7F** illustrates that DLD is significantly associated with Treg cells, Th2 cells, and NK cells. We also provide a correlation matrix between immune cells and functions in **Figure S6**. These results further confirmed the three hub genes are related to the immune infiltration microenvironment.

### Identification of drug candidates

**Figure 8:**
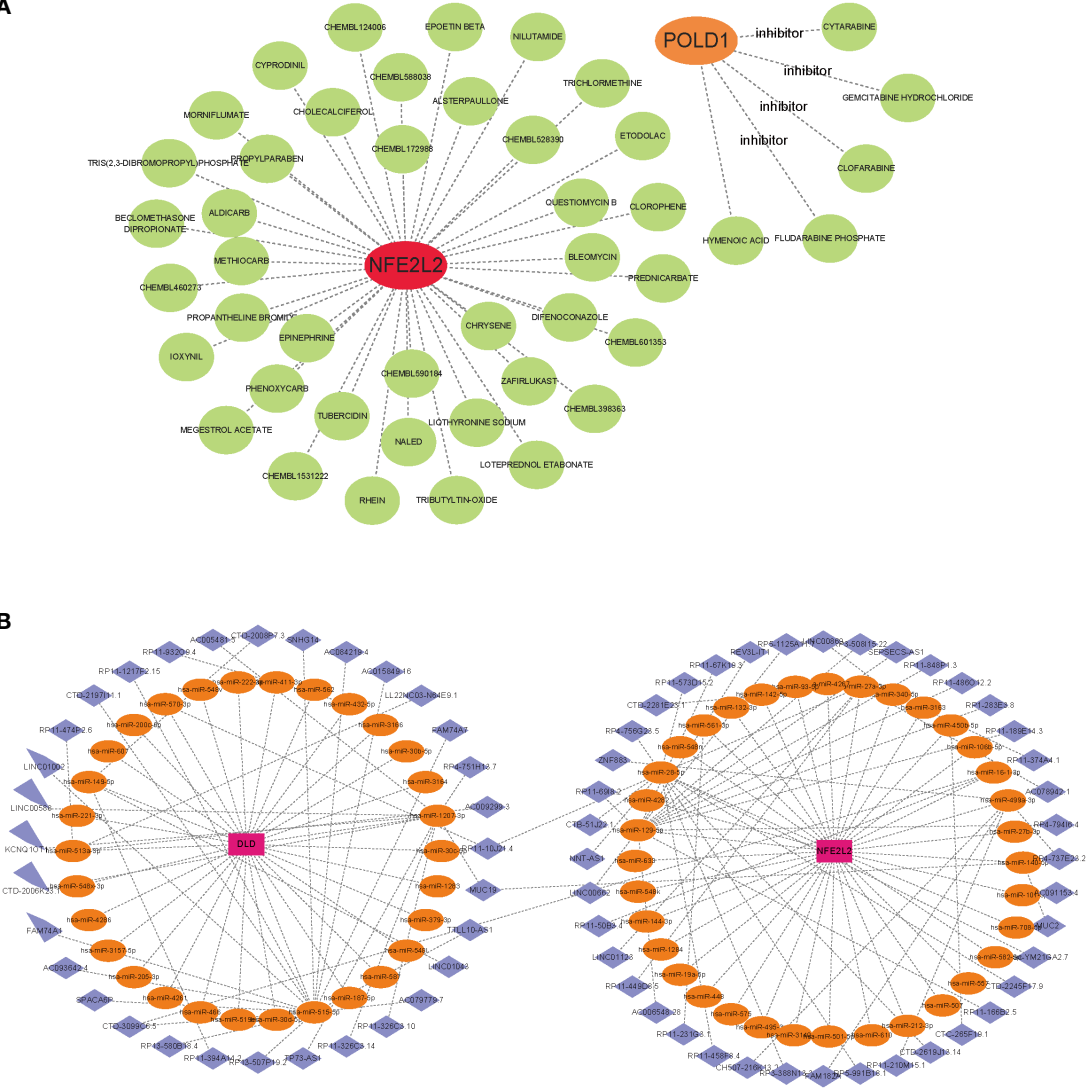
Gene-targeted drugs and ceRNA networks. **(A)** mRNA-drugs interaction network. The green circular node represented the drugs. **(B)** The ceRNA network based on marker genes. With Pink dots for mRNA, orange dots for miRNA, and blue dots for lncRNA.

To promote the development of future NAFLD treatment, the interaction relationship between hub genes and drugs was analyzed through DGIdb. Cytoscape analysis demonstrated the interaction between gene markers and drugs (**Figure 8A**). A total of 45 gene target drugs were enrolled: 40 for NFE2L2 and 5 for POLD1. However, no targeted drugs for DLD were predicted. Additionally, drug target predictive analysis was performed using DSigDB. Resveratrol, fumaric acid, and esculetin had the highest combined scores and were found to target the hub genes. Certain drugs, such as beclomethasone, rhein, chrysene, and those targeting NFE2L2, were identified in both DGIdb and DSigDB.

### CeRNA networks based on marker genes

Based on the three marker genes, a ceRNA network was developed using TargetScan, miRanda, and miRDB databases. A total of 3 mRNAs, 65 miRNAs, and 75 lncRNAs were identified (**Figure 8B**). The results indicated that 32 lncRNAs could regulate the expression of DLD through competitive binding of hsa-miR-1207-3p, hsa-miR-515-5p, and so on. Among these, 15 shared lncRNAs could target hsa-miR-515-5p. A total of 43 lncRNAs could competitively bind 35 miRNAs, such as hsa-miR-28-5p, hsa-miR-129-5p, hsa-miR-499a-3p, hsa-miR-27a-3p, and regulated NFE2L2. Among them, 14 and 10 lncRNAs target hsa-miR-129-5p and hsa-miR-28-5p, respectively. Additionally, hsa-miR-1207-3p and hsa-miR-140-5p could simultaneously bind lncRNA MJC19.

### Altered expression of CRGs in NAFLD

**Figure 9:**
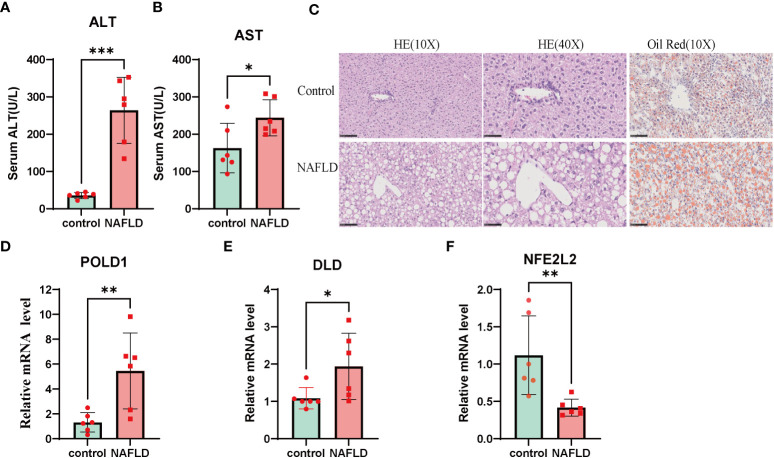
NAFLD mice model construction and Altered expression of CRGs in NAFLD. **(A, B)** serum alanine aminotransferase (ALT) and aspartate aminotransferase (AST) value in both wildtype and NAFLD mice. **(C)** The HE and Oil Red staining in wildtype and NAFLD mice. The result of HE and Oil red staining showed numerous fat droplets deposition in NAFLD group. **(D-F)** mRNA expression of DLD, POLD1, NFE2L2 by RT-PCR. Liver tissues and blood of the wildtype and NAFLD mice were collected. p values were showed as: *, p < 0.05; **, p < 0.01; ***, p < 0.001. CRGs, cuproptosis-related genes. Wild-type control mice(n=8), NAFLD mice(n=8).

The results of AST and ALT measurements exhibited a significant increase in the NAFLD group compared with the control group (**Figures 9A, B**). In addition, H&E and oil red O staining demonstrated substantial lipid deposition in the liver tissues of the NAFLD group, characterized by the formation of numerous fat droplets (**Figure 9C**). Taken together, these results collectively indicate the successful establishment of the NAFLD model.

To further investigate the role of the cuproptosis gene in NAFLD, the mRNA levels of three CRG hub genes were assessed using qRT-PCR. The results indicated that the expression of POLD1 and DLD was significantly altered in the NAFLD group compared with the control group (**Figures 9D–F**). These results indicated that CRGs may play a vital role in the development of NAFLD, thus further validating their potential regulatory role in NAFLD progression.

## Discussion

Due to the increasing prevalence of obesity and weight-related metabolic comorbidities, NAFLD has become one of the most common chronic liver diseases around the world (34). Late-stage NAFLD can progress to cirrhosis and hepatocellular carcinoma. Despite extensive research, the pathogenesis mechanism of NAFLD is complex and not fully understood (35), which has resulted in a lack of effective treatment drugs, reliable noninvasive diagnostic tools, and dynamic biomarkers (36). Therefore, identifying a validated biomarker for NAFLD is crucial for developing individual treatment strategies. Cuproptosis, a newly revealed mechanism of copper-dependent cell death, has proven to play an important role in Alzheimer’s disease, Crohn’s disease, and acute myocardial infarction (9, 11, 37–39). However, the specific pathogenesis and regulation of cuproptosis in NAFLD are not well understood. Therefore, our study aimed to investigate the diagnostic and prognostic values of CRGs in NAFLD pathogenesis, identify potential hub genes, and explore potential target drugs and ceRNA networks.

In the present study, we systematically investigated the differential expression of CRGs between NAFLD and control liver samples enrolled from the GEO database, and we finally identified nine DEGs related to cuproptosis. The difference in CRGs between NAFLD and the control liver sample indicated that CRGs may participate in the occurrence and progression of NAFLD. DE-CRGs correlation analysis indicated a relatively close correlation between DE-CRGs. However, at the protein level, only PDHB, ACO2, PDHA1, and DLD achieved a close correlation relationship. The results indicated a heterogeneous interaction of CRGs at the gene and protein levels.

GO and KEGG enrichment analyses indicated CRGs were significantly involved in the TCA pathway. The finding is somewhat consistent with that of Tsvetkov et al (11), who revealed that cuproptosis occurs by interfering with the TCA cycle by biding the copper to lipoylated components. However, our results indicated that FDX1 did not exhibit a difference between NAFLD and control liver samples, unlike Tsvetkov’s findings that FDX1 is a key regulator in cuproptosis. Our results suggest that the influence of cuproptosis on the TCA cycle of NAFLD may occur through the DE-CRGs identified in our study. Therefore, more researches are warranted to elucidate the relationship between CRGs and the TCA cycle for NAFLD. Additionally, the MF of GO analysis revealed that nine DE-CRGs were associated with iron-sulfur cluster binding, which is consistent with previous studies demonstrating the importance of iron-sulfur cluster protein loss in cuproptosis (11).

Machine learning models, a branch of artificial intelligence, have been increasingly used in medical research, including disease diagnosis, prognosis, and treatment prediction (40–42). These models can predict disease characteristics from complex data and self-trained strategies, providing reliable results with a lower error rate (37, 43). In the present study, we employed three machine learning classifiers (LASSO, RF, and SVM-REE) to identify hub genes for the diagnosis of NAFLD on the basis of the expression profiles of nine DE-CRGs. After selecting the intersection of LASSO, RF, and SVM-REE, we identified three hub genes that accurately predict the risk of NAFLD with an AUC value of 0.704. Moreover, the external validation dataset further confirmed the its reliability (AUC= 0.970). The AUC values of DLD, POLD1, and NFE2L2 were all greater than 0.7 in the validation dataset. We also constructed a nomogram model, calibration curves, and DCA using DLD, POLD1, and NFE2L2, which further verified the predictive efficacy of diagnosis and the clinical value of this model. Therefore, we conclude that the three-gene model is a reliable and robust biomarker for predicting the diagnosis of NAFLD.

DLD is a mitochondrial enzyme that oxidizes dihydrolipoamide to lipoamide and plays a critical role in energy metabolism (44). The GSEA result indicated that DLD is involved in the ncRNA metabolic process, cytokine-cytokine receptor interaction, and fatty acid metabolism. GSVA analysis further showed that high DLD expression participated in the process of energy metabolism, such as glycosaminoglycan degradation, and galactose metabolism. DLD participates in the decarboxylation process of pyruvate, leading to the formation of acetyl coenzyme A within the tricarboxylic acid (TCA) cycle. During its redox activity, it has the potential to generate reactive oxygen species (ROS) (45). The overproduction of ROS hampers the effectiveness of other antioxidant defense mechanisms in NAFLD, leading to heightened oxidative harm (46). Moreover, we found that the expression of DLD for NAFLD in the validation dataset was higher than that in the normal liver group. In addition, the mRNA expression of DLD in NAFLD mice was higher than that in the control group. However, further validation at the protein level is required to provide additional details in future. NFE2L2 is a transcription factor in antioxidative stress and modulating xenobiotics (47). Oxidative stress is one of the causes of NAFLD progression, and NFE2L2 has been shown to reduce reactive oxygen species production, thereby alleviating NAFLD (48). Studies have demonstrated that NFE2L2 knockout mice develop more severe steatosis and experience higher levels of oxidative stress than wild-type mice (49). In our study, we observed that the expression of NFE2L2, also known as nrf2, was lower in NAFLD samples than that in control liver samples, indicating a negative correlation between NFE2L2 expression and NAFLD. GSEA and GSVA analysis showed that NFE2L2 participated in some inflammation and immune processes such as JAK-STAT signaling, interleukin-1β, and inflammatory response pathway. In contrast, previous studies indicated that NFE2L2 regulated inflammation by NF-κB, interleukin-1β, and JNK pathway (49, 50). Nrf2 play a dual role in NAFLD, it serves as a mediator for the interaction between lipid metabolism and antioxidant defense mechanisms in NAFLD (**Figure S7**) (51). POLD1 encodes the catalytic subunits of DNA polymerase delta and plays a vital role in DNA damage repair and replication (52). Enrichment analysis further confirmed that the POLD1 is involved in the base excision repair, and DNA replication. Mutations in POLD1 have been linked to several diseases, such as colorectal cancer, breast cancer, mandibular hypoplasia–deafness–progeroid syndrome (MDP), and Alzheimer’s disease (53–56). Furthermore, hepatocellular carcinoma, the end-stage of NAFLD, displays higher POLD1 expression levels than normal liver tissues, and this is associated with a poor prognosis (57). Our study found that POLD1 was overexpressed in NAFLD, indicating that POLD1 variations may occur during hepatic precancerous lesions arising from NAFLD. All DLD, POLD1, and NFE2L2 were found to be associated with the cytokine-cytokine receptor interaction pathway. This pathway, which includes the TGF-β family and TNF family, plays a crucial role in NAFLD as an essential component of the inflammatory process (58, 59).

NAFLD has a complex etiology involving several factors, and recent evidence suggests that immunity plays a critical role in the pathogenesis of this condition (60–62). In our study, we employed the CIBERSORT algorithm and ssGSEA to analyze immune infiltration in NAFLD. The ratios of Macrophages, Mast cells, NK cells, Tregs cell, resting Dendritic cells, and TIL was higher in NAFLD compared with control liver samples. Macrophage, populated in the liver during NAFLD, express a high level of Spp1, Cd9, and Trem2 and participate in hepatic fibrosis (63–65). Mast cells are important components of the innate immune system and have been indicated to accumulate in the liver during hepatic injuries and fibrosis (66). Mast cells also populate bile duct-ligated (BDL) mice models and reside near injured ducts (67–69). Inhibition or genetic loss of MCs in BDL and Mdr2^-/-^ mice reduces biliary senescence, and liver fibrosis, and alter the progressive course of NAFLD (68, 70). Mast cells have also been shown to mediate biliary senescence and promote ductular reaction during NAFLD (69). Furthermore, our results suggest that POLD1 is closely correlated with many immune cells and immune functions, which is consistent with previous research findings (71). Overall, these results indicate the crucial role of immunity in the development of NAFLD, and suggest that POLD1 may play an essential role in the immune microenvironment of NAFLD patients. In addition, we found that both DLD and NEF2L2 were significant correlated with Treg cells, which play a multifaceted role in NAFLD. On one hand, Treg cells promote fibrosis by releasing TGF-β. On the other hand, they inhibit fibrosis through the secretion of IL-10 (72). This finding suggests that DLD and NEF2L2 may play a vital role in immune microenvironment of NAFLD.

Owing to the high potential of cuproptosis-targeted therapeutic agents, our study analyzed gene-targeted drugs that focused on the three marker genes. Among the gene-targeted therapeutic agents, rhein has various pharmacological effects, such as hepatoprotective effects and anti-inflammation effects (73, 74). Previous studies have indicated that rhein significantly reduces serum levels of AST, ALT, and GLU in NAFLD rats and alleviates liver structure and dysfunction (75). LncRNAs, as competitive endogenous RNAs, can compete with binding miRNA to regulate the expression of mRNA, thus affecting the physical activities of different cells (76, 77). Considering the potential relevance of the lncRNA–miRNA–mRNA pathway, we constructed a ceRNA network for NAFLD. Our results indicate that lncRNAs can regulate the expression of two CRGs (NFE2L2 and DLD). In this way, gene-targeted drugs and ceRNA network analysis provide a new horizon for the in-depth study of drug selection and NAFLD pathogenesis. However, further validation is necessary to confirm specific details because of a lack of relevant in vitro and in vivo research.

Nevertheless, our study has some limitations that need to be acknowledged. First, the data in this study were obtained from the GEO public database, which may introduce selection bias caused by the lack of raw sequencing data. Second, although mouse experiments somewhat confirmed the result of bioinformatics analysis, we cannot obtain enough clinical samples of NAFLD within a limited time frame, and more clinical samples need to be enrolled to clarify the underlying mechanisms of cuproptosis in NAFLD. Third, the discrepancy between the RAN sequence and qRT-PCR results suggests a complex regulatory mechanism of cuproptosis in NAFLD. We could not explore the regulatory mechanism of cuproptosis in NAFLD within a limited time frame in the present study. Hence, future investigations in the future are strong needed.

## Conclusion

In conclusion, our study uncovered an association between CRGs and infiltrated immune cells, highlighting the significant heterogeneity of immune response between NAFLD patients and control liver samples. Using a machine learning model, we identified a three-CRG–based signature that can accurately diagnose NAFLD patients. Our findings offer novel insights into the role of cuproptosis in NAFLD and provide a better understanding of the underlying pathogenesis mechanism and potential therapeutic targets for NAFLD.

## Data availability statement

The original codes used for analyses presented in the study are publicly available. This data can be found here: https://github.com/ouyan1990/NAFLD-cuproptosis.

## Ethics statement

All animal experiments were approved by the Animal Care and Use Committee of Guangxi Medical University.

## Author contributions

The formal analysis and original draft of the manuscript were conducted by GO and ZW. Project administration was performed by ZL, YW, and TL. Software analysis was carried out by GO, ZW, ZL, ZWW, and JL. Data curation was conducted by GO, GH, GP, and JG. The experiment was performed by ZL and YZ. The writing, reviewing, and editing of the article were contributed by GO, YZ, GY, and SH. Funding acquisition was provided by GO, ZWW, GY, and SH. All authors contributed to the article and approved the final version for submission.
