# Supplementary material for: Identification and validation of potential diagnostic signature and immune cell infiltration for NAFLD based on cuproptosis-related genes by bioinformatics analysis and machine learning
Source: Front Immunol. 2023 Sep 26;14:1251750. doi: 10.3389/fimmu.2023.1251750 (PMC10562635; doi:10.3389/fimmu.2023.1251750)
Supplement: Supplementary file 1 [file DataSheet_1.docx]

**­­Identification and validation of potential diagnostic signature and immune cell infiltration for NAFLD based on** **cuproptosis-related genes by bioinformatics analysis and machine learning**

Guoqing Ouyang^1,2,3,4,#^, Zhan Wu^1,2,3,#^, Zhipeng Liu^1,2,3,#^, Guandong Pan^4,5^, Yong Wang^1,2,3^, Jing Liu^1,2,3^, Jixu Guo^1,2,3^, Tao Liu^6^, Guozhen Huang^1,2,3^, Yonglian Zeng^1,2,3,^, Zaiwa Wei^1,2,3,^*, Songqing He^1,2,3,^*, [Guandou Yuan](https://pubmed-ncbi-nlm-nih-gov-443--bjmu.jitui.me/?term=Yuan+G&cauthor_id=35069557)^1,2,3,^*

^1^Division of Hepatobiliary Surgery, The First Affiliated Hospital of Guangxi Medical University, Nanning, Guangxi 530021, China.

^2^Key Laboratory of Early Prevention and Treatment for Regional High Frequency Tumor (Guangxi Medical University), Ministry of Education, Nanning, Guangxi 530021, China.

^3^Guangxi Key Laboratory of Immunology and Metabolism for Liver Diseases, Nanning, Guangxi 530021, China.

^4^Liuzhou Key Laboratory of Liver Cancer Research, Liuzhou People’s Hospital, Liuzhou, Guangxi 545001, China.

^5^Liuzhou Hepatobiliary and Pancreatic Diseases Precision Diagnosis Research Center of
Engineering Technology, Liuzhou, Guangxi 545001, China.

^6^Department of general surgery, Luzhai People’s Hospital, Liuzhou, Guangxi 545001, China.

^#^ These authors have contributed equally to this work

*Correspondence:

Songqing He, Division of Hepatobiliary Surgery, The First Affiliated Hospital of Guangxi Medical University, NO 6 Shuangyong Road, Nanning 530021, Guangxi, China. Email: dr_hesongqing@163.com

Zaiwa Wei, Division of Hepatobiliary Surgery, The First Affiliated Hospital of Guangxi Medical University, NO 6 Shuangyong Road, Nanning 530021, Guangxi, China. E-mail: 875828696@qq.com

Guandou Yuan, Division of Hepatobiliary Surgery, The First Affiliated Hospital of Guangxi Medical University, NO 6 Shuangyong Road, Nanning 530021, Guangxi, China. E-mail: dr_yuangd@gxmu.edu.cn

**Contents**

**[Supplementary tables 2](#_Toc14831)**

[Table S1 the primers of GAPDH, POLD1, NFE2L2, and DLD 3](#_Toc18861)

[Table S2 The correlation between 3 cuproptosis genes and immune cells 3](#_Toc24535)

**[Supplementary Figures 6](#_Toc9819)**

[Figure S1 7](#_Toc27705)

[Figure S2 7](#_Toc28947)

[Figure S3 8](#_Toc14100)

[Figure S4 8](#_Toc8080)

[Figure S5 9](#_Toc19983)

[Figure S6 10](#_Toc1132)

[Figure S7 10](#_Toc28348)

**Supplementary tables**

**Table S1** the primers of GAPDH, POLD1, NFE2L2, and DLD

| **Genes** | | **Sequences (5’-3’)** |
| --- | --- | --- |
| **GAPDH** | Forward | CCCACTAACATCAAATGGGG |
|  | Reverse | CCTTCCACAATGCCAAAGTT |
| **POLD1** | Forward | TGCTGATGCCTGTGGTGAAGAC |
|  | Reverse | AGGAGAAGTCCAGGGTGGCAAT |
| **NFE2L2** | Forward | AAGATGCCTTGTACTTTGAAGACTGT |
|  | Reverse | GGAAAATAGCTCCTGCCAAACTT |
| **DLD** | Forward | GGGTTGGCAAATCGGAAGAACAG |
|  | Reverse | TCACCATGCCATCTGTGTCAGC |

**Table S2** The correlation between 3 cuproptosis genes and immune cells

| **Gene** | **Immune** | **cor** | **pvalue** |
| --- | --- | --- | --- |
| DLD | B cells naive | -0.30658 | 0.0028 |
| POLD1 | B cells naive | -0.18175 | 0.081234 |
| NFE2L2 | B cells naive | -0.15423 | 0.139915 |
| DLD | B cells memory | -0.01278 | 0.903258 |
| POLD1 | B cells memory | 0.159289 | 0.127233 |
| NFE2L2 | B cells memory | 0.009713 | 0.926373 |
| DLD | Plasma cells | 0.307181 | 0.002744 |
| POLD1 | Plasma cells | -0.23346 | 0.02431 |
| NFE2L2 | Plasma cells | 0.185904 | 0.074403 |
| DLD | T cells CD8 | -0.14185 | 0.174984 |
| POLD1 | T cells CD8 | 0.129601 | 0.21566 |
| NFE2L2 | T cells CD8 | -0.36276 | 0.000352 |
| DLD | T cells CD4 naive | 0.167601 | 0.108317 |
| POLD1 | T cells CD4 naive | -0.19676 | 0.058706 |
| NFE2L2 | T cells CD4 naive | 0.099851 | 0.340951 |
| DLD | T cells CD4 memory resting | 0.162718 | 0.119144 |
| POLD1 | T cells CD4 memory resting | 0.010429 | 0.920963 |
| NFE2L2 | T cells CD4 memory resting | 0.282483 | 0.00608 |
| DLD | T cells CD4 memory activated | 0.240064 | 0.020461 |
| POLD1 | T cells CD4 memory activated | -0.28325 | 0.005937 |
| NFE2L2 | T cells CD4 memory activated | 0.397502 | 7.97E-05 |
| DLD | T cells follicular helper | -0.22771 | 0.028153 |
| POLD1 | T cells follicular helper | -0.04648 | 0.658193 |
| NFE2L2 | T cells follicular helper | -0.05133 | 0.62512 |
| DLD | T cells regulatory (Tregs) | -0.25749 | 0.012711 |
| POLD1 | T cells regulatory (Tregs) | 0.183301 | 0.078626 |
| NFE2L2 | T cells regulatory (Tregs) | -0.44638 | 7.30E-06 |
| DLD | T cells gamma delta | 0.467534 | 2.30E-06 |
| POLD1 | T cells gamma delta | -0.23182 | 0.025361 |
| NFE2L2 | T cells gamma delta | 0.260696 | 0.011607 |
| DLD | NK cells resting | -0.04608 | 0.660918 |
| POLD1 | NK cells resting | 0.090858 | 0.38641 |
| NFE2L2 | NK cells resting | -0.01227 | 0.907048 |
| DLD | NK cells activated | -0.19591 | 0.05983 |
| POLD1 | NK cells activated | 0.083519 | 0.426075 |
| NFE2L2 | NK cells activated | -0.37537 | 0.000209 |
| DLD | Monocytes | -0.2477 | 0.016674 |
| POLD1 | Monocytes | 0.106789 | 0.308287 |
| NFE2L2 | Monocytes | 0.090787 | 0.386783 |
| DLD | Macrophages M0 | -0.00716 | 0.9457 |
| POLD1 | Macrophages M0 | 0.029097 | 0.78188 |
| NFE2L2 | Macrophages M0 | -0.01778 | 0.865701 |
| DLD | Macrophages M1 | -0.14302 | 0.171441 |
| POLD1 | Macrophages M1 | 0.291476 | 0.004587 |
| NFE2L2 | Macrophages M1 | -0.00082 | 0.993771 |
| DLD | Macrophages M2 | 0.039935 | 0.703899 |
| POLD1 | Macrophages M2 | 0.134538 | 0.198172 |
| NFE2L2 | Macrophages M2 | -0.25871 | 0.01248 |
| DLD | Dendritic cells resting | 0.109565 | 0.295805 |
| POLD1 | Dendritic cells resting | 0.011493 | 0.912937 |
| NFE2L2 | Dendritic cells resting | -0.06696 | 0.523646 |
| DLD | Dendritic cells activated | -0.14238 | 0.173365 |
| POLD1 | Dendritic cells activated | -0.21705 | 0.036629 |
| NFE2L2 | Dendritic cells activated | -0.00189 | 0.985648 |
| DLD | Mast cells resting | 0.307164 | 0.002746 |
| POLD1 | Mast cells resting | 0.216217 | 0.037378 |
| NFE2L2 | Mast cells resting | -0.0199 | 0.849827 |
| DLD | Mast cells activated | -0.34138 | 0.000811 |
| POLD1 | Mast cells activated | -0.06471 | 0.537754 |
| NFE2L2 | Mast cells activated | 0.048735 | 0.64272 |
| DLD | Eosinophils | -0.13588 | 0.194062 |
| POLD1 | Eosinophils | -0.01535 | 0.88392 |
| NFE2L2 | Eosinophils | 0.046214 | 0.660022 |
| DLD | Neutrophils | 0.032986 | 0.753608 |
| POLD1 | Neutrophils | -0.17103 | 0.101177 |
| NFE2L2 | Neutrophils | 0.194634 | 0.061551 |

**Supplementary Figures**

**Figure S1**


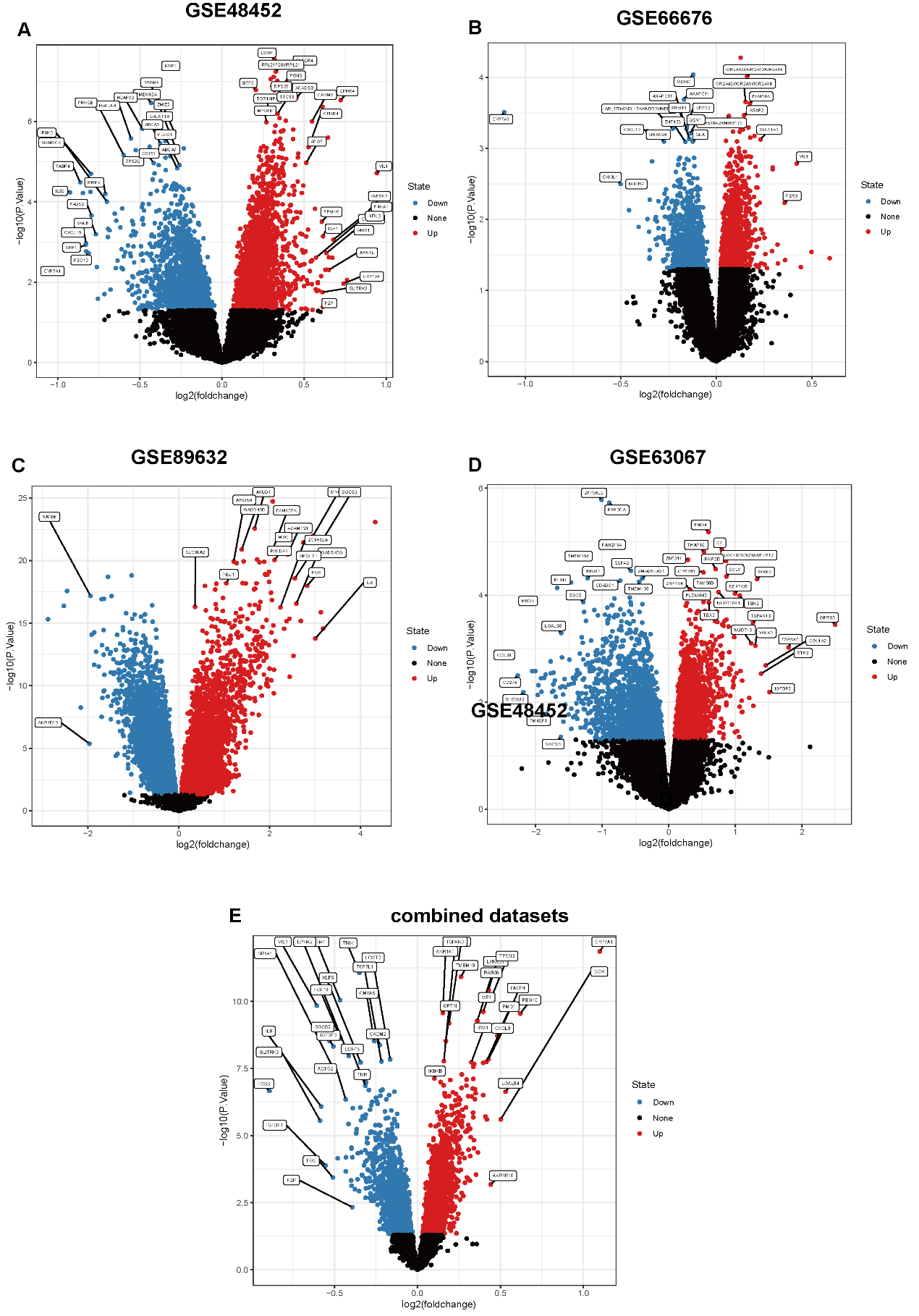


**Volcano plot of the differentially expressed genes (DEGs).** (A) The volcano map of GSE48452. (B) The volcano map of GSE66676. (C) The volcano map of GSE89632. (D) The volcano map of GSE63067. (E) The volcano map of the combined datasets. The over-expressed genes are marked in red and the down-expressed genes are marked in blue.

**Figure S2**


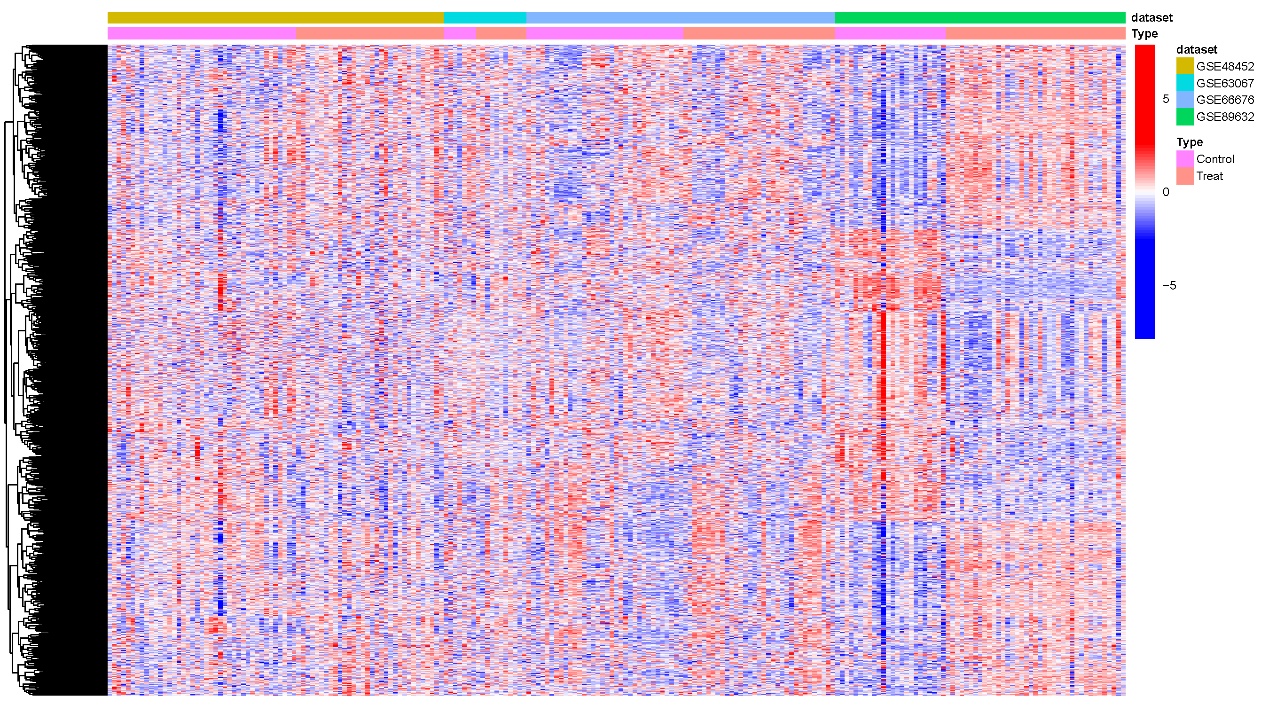


The expression patterns of 4,170 DEGs were presented in the heatmap between NAFLD and control. DEG: differential expression genes.

**Figure S3**


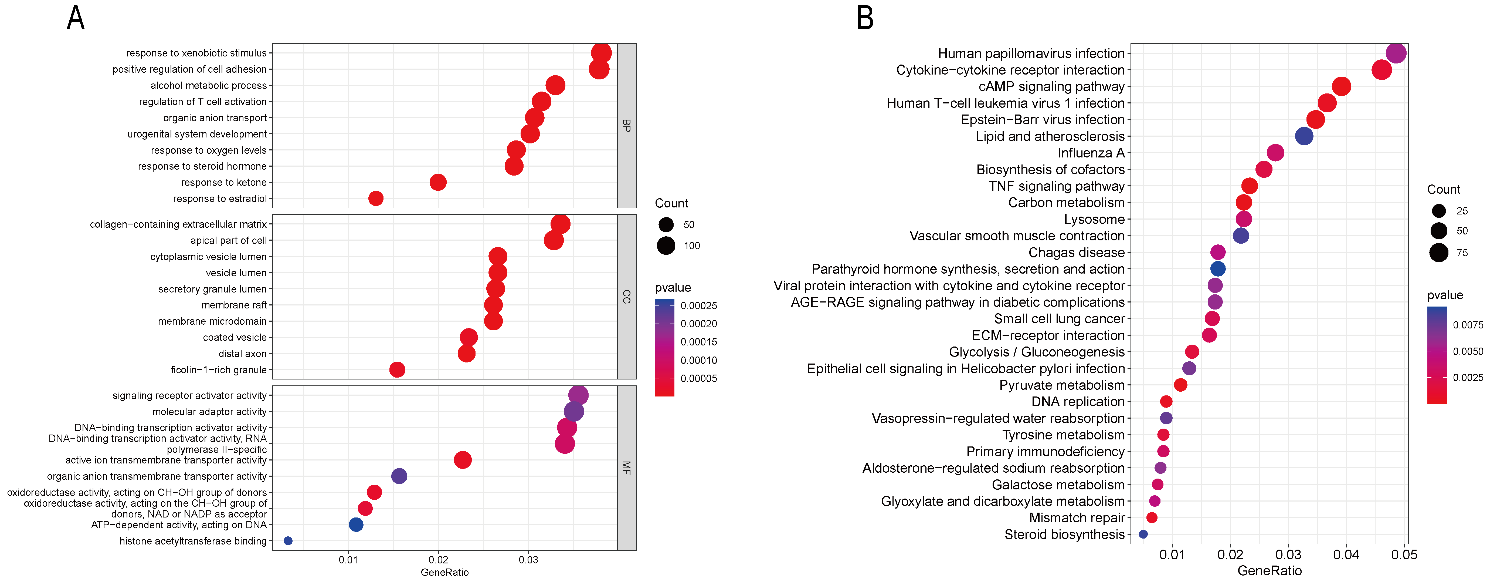
**Functional analyses for the 4,170 DEGs.** (A) Bubble diagrams of the GO enrichment analysis of 4,170 DEGs. (B) Bubble diagrams of the KEGG enrichment analysis of 4,170 DEGs. DEGs, differentially expressed genes.

**Figure S4**


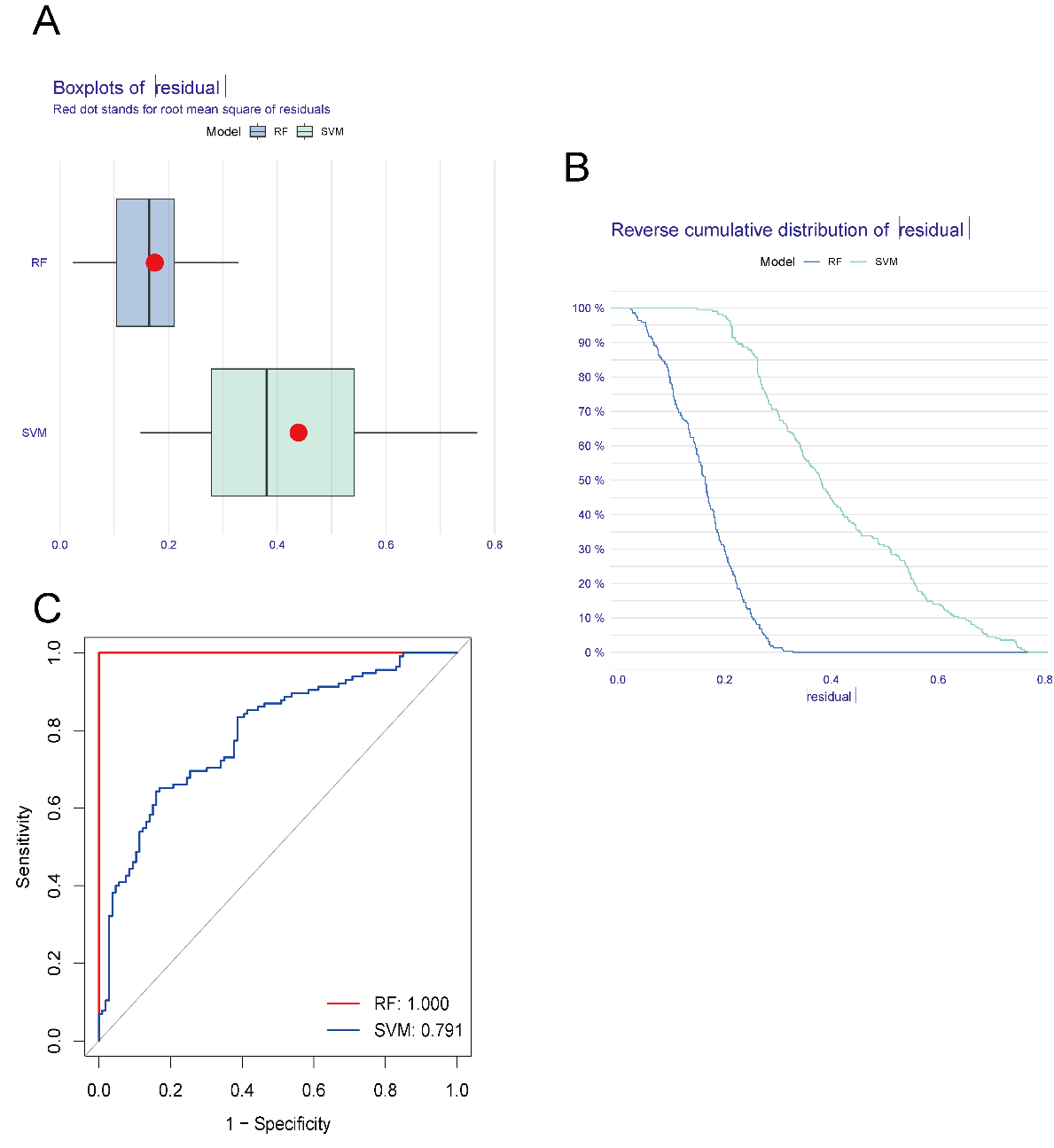


**Cumulative residual distributions and ROC results of RF and SVM.** (A)Boxplots of residual values of RF (Random Forest) and SVM (Support Vector Machine) models. Red dot represents the mean residual value. (B) Cumulative residual distribution plots of RF and SVM models. (C) ROC results of the RF and SVM.

**Figure S5**


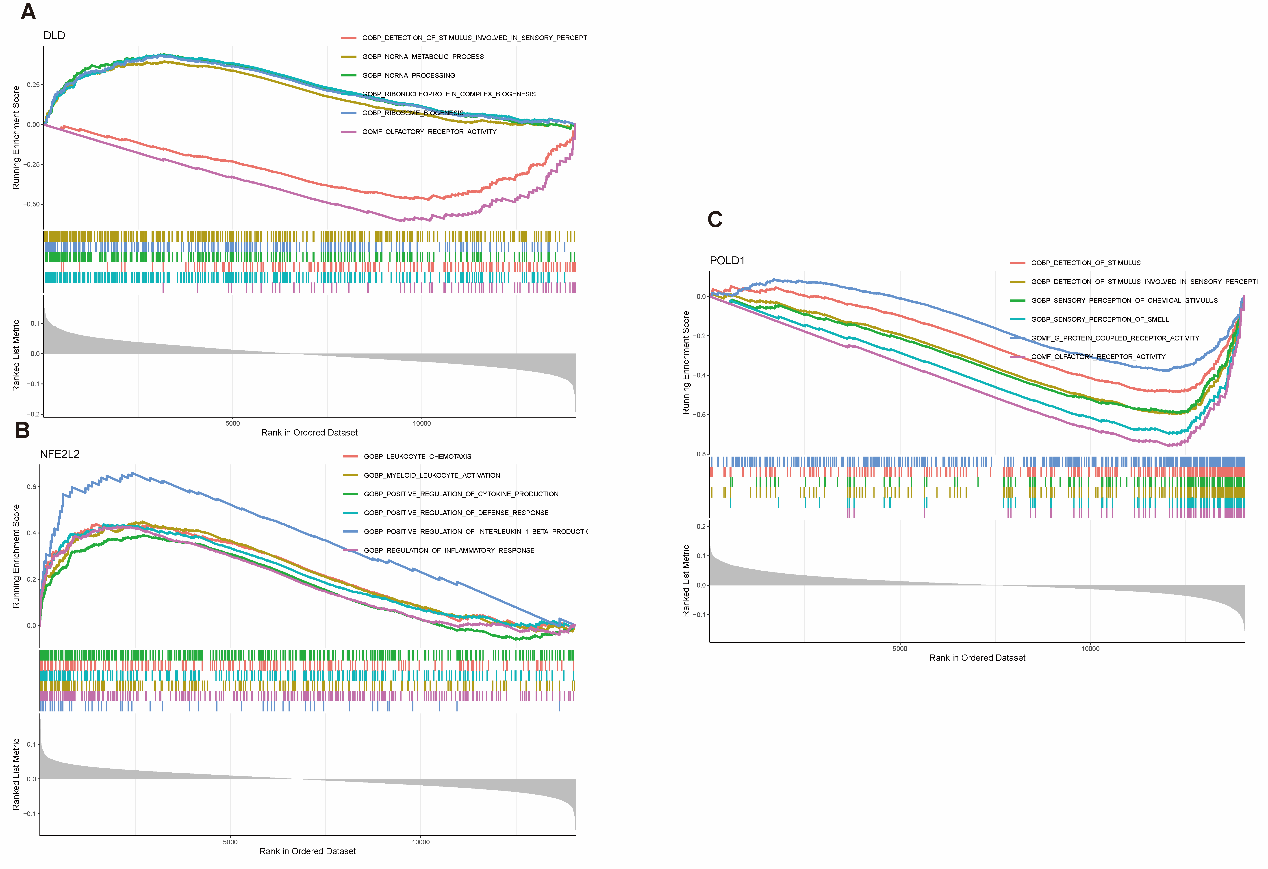
 GSEA result of GO enrichment for DLD(A), NFE2L2(B), and POLD1(C).

**Figure S6**


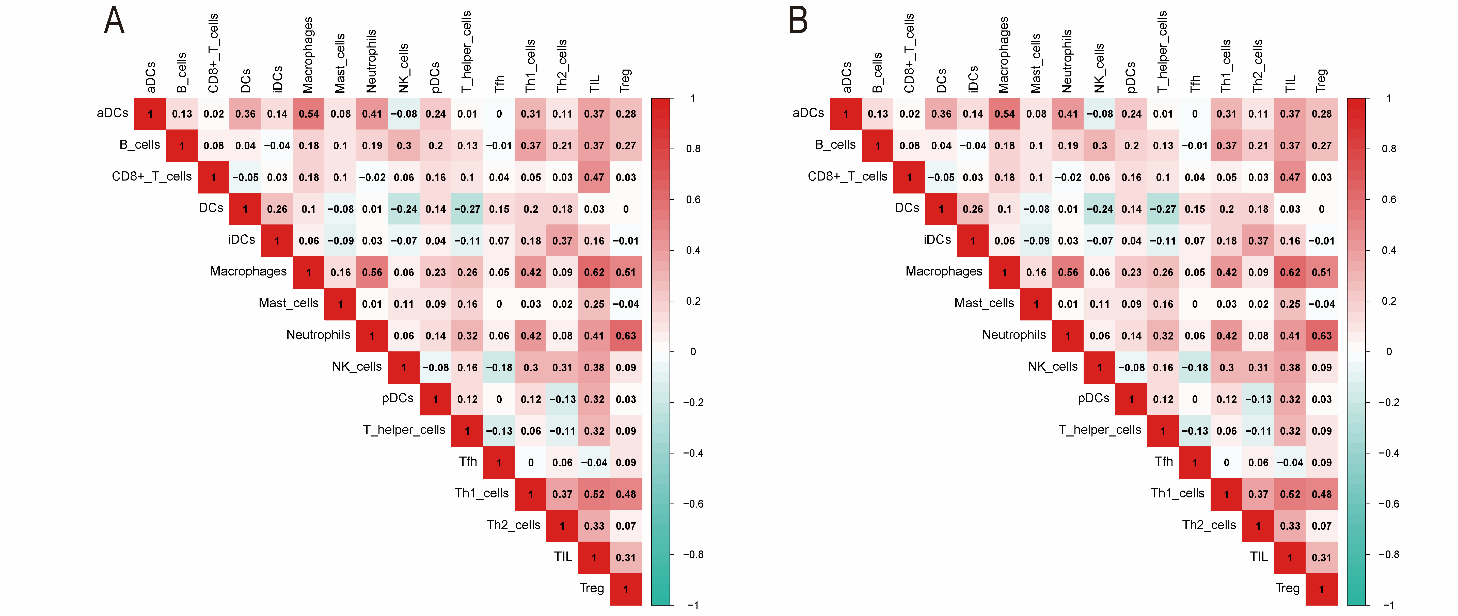


The correlation matrix between immune cells and functions(A-B)

**Figure S7**


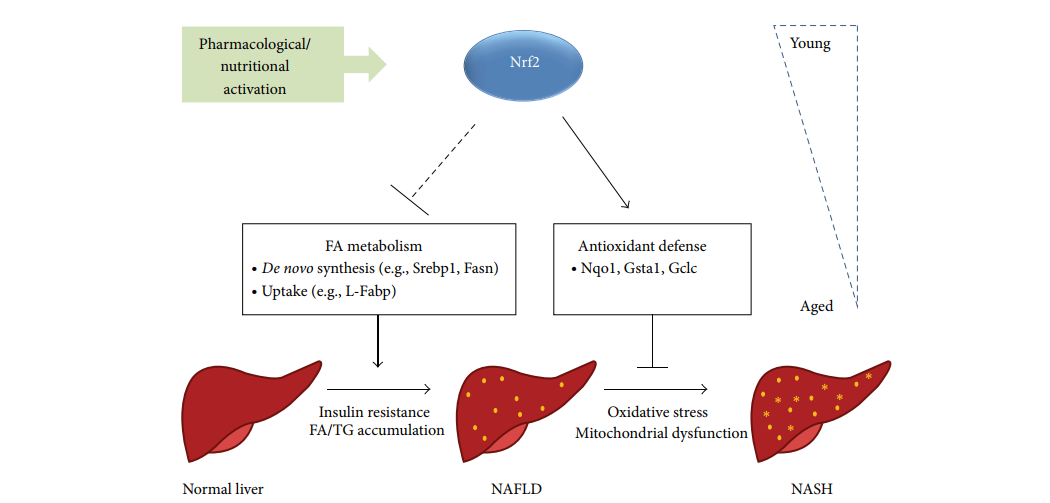


**Schematic summary of the proposed protective roles of Nrf2 in nonalcoholic fatty liver disease (NAFLD).** The progression from the simple accumulation of lipids in the hepatocyte to steatohepatitis (NASH) is presented NASH is linked with inflammatory processes, fibrosis, and eventual cirrhosis. The traditional perspective suggests that Nrf2 orchestrates the removal of reactive oxygen species (ROS) and electrophiles stemming from lipid peroxidation, thereby averting oxidative stress and mitochondrial dysfunction in hepatocytes. Furthermore, an expanding body of literature indicates that Nrf2 exerts control over fatty acid metabolism by inhibiting genes that contribute to hepatic lipid accumulation. Experimental evidence in rodent models showcases the age-dependent suppression of steatohepatitis through both mechanisms, attainable through either pharmacological means (e.g., CDDO-Im) or nutritional interventions (e.g., sulforaphane) to induce Nrf2 activation [1].

**Reference:**

1. Chambel SS, Santos-Gonçalves A, Duarte TL. The Dual Role of Nrf2 in Nonalcoholic Fatty Liver Disease: Regulation of Antioxidant Defenses and Hepatic Lipid Metabolism. Biomed Res Int. 2015;2015:597134. doi: 10.1155/2015/597134.
